# Benefit finding in long-term prostate cancer survivors

**DOI:** 10.1007/s00520-020-05971-3

**Published:** 2021-01-15

**Authors:** Irène Lassmann, Andreas Dinkel, Birgitt Marten-Mittag, Matthias Jahnen, Helga Schulwitz, Jürgen E. Gschwend, Kathleen Herkommer

**Affiliations:** 1 Department of Urology, Klinikum rechts der Isar, School of Medicine, Technical University of Munich, Ismaninger Str. 22, 81675 Munich, Germany; 2 Department of Psychosomatic Medicine and Psychotherapy, Klinikum rechts der Isar, School of Medicine, Technical University of Munich, Langerstr. 3, 81675 Munich, Germany

**Keywords:** Prostate cancer, Urological malignancy, Cancer survivor, Benefit finding, Posttraumatic growth

## Abstract

**Purpose:**

Benefit finding (BF) represents possible positive changes that people may experience after cancer diagnosis and treatment and has proven to be valuable to the psychological outcome. Knowledge of such beneficial consequences of prostate cancer (PCa) is limited in long-term survivors (> 5 years). Thus, the present study investigated the occurrence of benefit finding (BF) and its determinants in a large sample of (very-) long-term PCa survivors.

**Methods:**

BF was assessed in 4252 PCa survivors from the German database “Familial Prostate Cancer” using the German version of the Benefit Finding Scale (BFS). Associations between BF and sociodemographic, clinical, and psychosocial (e.g., depressive and anxiety symptoms and perceived severity of the disease experience) variables were analyzed using hierarchical multiple linear regression analysis.

**Results:**

Mean age at survey was 77.4 years (SD = 6.2) after a mean follow-up of 14.8 years (SD = 3.8). Mean BFS score was 3.14 (SD = 1.0); the prevalence of moderate-to-high BF (score ≥ 3) was 59.7%. Younger age at diagnosis, lower educational level, and higher perceived severity of the disease experience were predictive of BF. Objective disease severity or family history of PCa was not uniquely associated with BF.

**Conclusions:**

BF occurs in older, (very-) long-term PCa survivors. Our findings suggest that the self-asserted severity of the disease experience in a patient’s biography is linked to BF in the survivorship course above all tangible sociodemographic and clinical factors.

**Implications for cancer survivors:**

PCa survivors may express BF regardless of clinical disease severity. Treating urologists should consider inquiring BF to enrich a patient’s cancer narrative.

## Introduction

Reevaluation of life and perception of positive changes after a stressful life event such as cancer have commonly been defined as benefit finding (BF) or posttraumatic growth (PTG) [1]. Reported positive outcomes may include an increased appreciation and acceptance of life, shifts in interpersonal relationships (family/friends), spiritual growth, strengthening of one’s personality, or improvement in health behaviors [2]. Both concepts, BF and PTG, share relevant features, and thus, these terms are often used interchangeably [1, 3–5]. Studies that investigated the relation among both constructs found a strong association, e.g., *r* = 0.71 in the study by Jansen et al. [6]. Moreover, metaanalyses investigating whether the results were moderated by measures assessing BF vs. PTG did not find an effect, suggesting that associations are largely comparable for BF and PTG. This metaanalytic evidence revealed as well that BF and PTG showed a small positive association with posttraumatic stress [5, 7]. Furthermore, small associations were reported for enhanced positive well-being, while the association between BF or PTG and negative mental health as well as subjective physical health remains inconclusive [1, 3]. Associations with objective physical health–related outcomes are also inconclusive; decreased stress–related biomarkers (i.e., healthier diurnal cortisol pattern and lymphocytes proliferation) have been described singularly [8, 9]. However, improved global outcomes (e.g., reduced recurrence rates) have not been associated with BF and PTG. Regarding determinants of positive change after critical events, frequently though not unanimously reported sociodemographic characteristics include younger age at disease onset, female gender, and non Caucasian ethnicity in ethnic diverse study populations [1]. The objective severity of the disease has occasionally been linked to the perception of benefits. In a mixed sample of 83 cancer patients, Lechner et al. [4] described a curvilinear association between BF level and tumor stage. Moreover, higher perceived stress, perceived poor health, and perceived threat or burden arising from the disease was associated with BF and PTG [6, 10–12]. Furthermore, active coping, positive reappraisal, social support, and optimism [1, 10, 13, 14] but also intrusive thoughts and social constraints [14] showed significant associations with such positive changes. Most studies focused on short-term cancer survivors (≤ 5 years). However, Jansen et al. [6] reported a prevalence rate of 46–64% for BF and PTG in colorectal cancer survivors at a 5-year follow-up, and Lelorain et al. [15] found similar levels of PTG in long-term breast cancer survivors (5–15 years after diagnosis) compared with short-term survivors.

Prostate cancer (PCa) is the most common noncutaneous malignancy and a leading cause of cancer-related deaths in men in most Western countries [16]. In Germany, the median age of affected individuals at diagnosis is 72 years. The long-term survival rate of PCa is 88% at 10-year follow-up, all stages combined [17]. Besides age and ethnicity, another known risk factor is a positive family history of PCa in approximately 20% of the cases [18]. PCa is diagnosed in 62% of cases in an organ-confined stage and can be treated with a curative intent [17]. However, 15–45% of patients experience biochemical recurrence (BCR) during follow-up: initially low or undetectable levels of prostate-specific antigen (PSA) after primary therapy will rise again, indicating probable local relapse or occurrence of metastasis. Even after 10 years of BCR-free survival, BCR will occur in approximately 10% of patients [19]. Hence, annual follow-up visits are recommended even in (very-) long-term survivors [20]. Furthermore, side effects from both primary treatment and androgen deprivation therapy can have a negative impact on the quality of life [21, 22]. Moreover, up to 20% of long-term PCa survivors experience some kind of cancer-related distress [23–25]. Therefore, PCa may be considered a chronic or longitudinal stressor.

BF and PTG have been reported in PCa survivors using active coping strategies and receiving social and emotional support [26–28]. However, these studies comprised only patients with short follow-up periods (up to 18 months posttreatment) or time since diagnosis was not reported. Besides these factors, clinical variables like BCR and family history might be associated with positive changes in life after PCa. Associations with such variables might be helpful for understanding and promoting personal growth in the survivorship trajectory, but have, to our knowledge, not yet been reported.

Thus, the present study investigated the occurrence of BF in a large sample of (very-) long-term PCa survivors and the associations of BF with sociodemographic, clinicopathological, and psychosocial characteristics.

## Methods

### Study design

This is a cross-sectional study conducted under the German nationwide research project “Familial Prostate Cancer”. This project has been recruiting patients with PCa since 1993. The project aims to identify genetic and exogenous risk factors for PCa and to compare the clinical course and survivorship of patients with and without a family history of PCa. Briefly, patients are recruited by treating urologists or rehabilitation clinics mostly after radical prostatectomy (RP), and written consent is obtained. Sociodemographic and family history data are gathered via self-reporting questionnaires; clinical and histopathological data are reported by each patient’s treating urologist. Mailed follow-up questionnaires, including questions to assess the current PSA level, a potentially ongoing treatment of the PCa and current family history of PCa, are sent to the patients annually. Additionally, various psychosocial aspects have been investigated in the past years [23, 24].

### Procedure

From October 2018 to November 2018, annual follow-up questionnaires, along with a prepaid return envelope, were sent to 6379 patients with known histopathological record and at least one returned follow-up questionnaire in the past. By June 2019, 4252 participants (67%) had returned the questionnaire.

### Measures

#### Sociodemographic and clinicopathological characteristics

Sociodemographic data included in this analysis were age at survey, partnership, educational level, and number of children. Clinical data included age at diagnosis, time since diagnosis, presence of a second primary cancer, family history of PCa (yes [at least one consanguine relative with PCa] vs. no), PSA level at diagnosis, histopathological Gleason score, histopathological grading, organ-confined stage at RP according to the TNM classification of 2002, type of primary treatment, and ongoing treatment for PCa at survey. Biochemical recurrence after RP was defined as PSA level of ≥ 0.2 ng/ml and was analyzed for the entire follow-up period and at survey.

#### Benefit finding

The main outcome variable Benefit finding (BF) was assessed using the German version of the 17-item Benefit Finding Scale (BFS). The original version of the BFS, developed for a breast cancer population, and its German translation have proven to be a valid and reliable instrument and have shown a unidimensional structure [29–31]. We adapted the BFS by modifying the original stem to “having had prostate cancer”. The stem is followed by 17 items describing potential beneficial consequences of the disease, that is, changes in interpersonal/familial relationships, acceptance of the deficiencies in life, or spiritual growth. Responses to each item are scored on a five-point Likert scale ranging from 1 (not at all) to 5 (extremely). [29] Strong endorsement of an item was defined as a score of ≥ 4 (quite a bit), as previously described [32, 33]. Prevalence of moderate-to-high BF was defined as a mean scale score of ≥ 3 (moderately), as previously described [6]. Cronbach’s alpha coefficient for this sample was 0.96, indicating high internal consistency.

#### Depressive and anxiety symptoms

Psychological distress was assessed using the validated ultra-brief instruments Patient Health Questionnaire-2 (PHQ-2) for depressive symptoms and Generalized Anxiety Disorder-2 (GAD-2) scale for symptoms of anxiety. For both scales, a cutoff score of ≥ 3 (range, 0–6) indicates a clinical level of symptom burden [34]. Cronbach’s alpha coefficients for the PHQ-2 and GAD-2 scales were 0.76 and 0.76, respectively, indicating satisfactory internal consistency.

#### Perceived severity of the disease experience

The perceived severity of the disease experience as a stressful life event was assessed with the item “Having had prostate cancer is one of the worst things that happened to me in my life” (adapted from [35]). The participants were asked to rate this statement on a 4-point Likert scale ranging from 1 (strongly disagree) to 4 (strongly agree). Agreement/disagreement with this statement was used as operationalization of perceived severity of the disease experience, ranging from none to high.

### Statistical analysis

Descriptive statistics were computed for all study variables. Prorating was used to impute up to 4 missing out of 17 items (23.5%) of the BFS using the mean score of the participant’s remaining items. Correlations between BF and psychosocial variables were reported as Pearson correlation coefficients. Hierarchical multiple regression analysis was conducted to predict BF via sociodemographic (step 1), clinical (step 2), and psychosocial (step 3) variables. All analyses were conducted using the Statistical Package for the Social Sciences, version 24 (IBM Corp., Chicago, IL, USA).

## Results

### Sample characteristics

The mean age of the 4252 PCa survivors who returned the questionnaire was *M* **=** 77.4 years (SD **=** 6.4). The participants were mostly living in a partnership (85.4%), and 88.4% of them had at least one child. Among all participants, 29.4% held an academic degree, whereas 41.3% had a low educational level. The mean time since diagnosis of PCa was *M* **=** 14.8 years (SD **=** 3.8), and approximately 8% had survived for more than 20 years; 38.5% had a positive family history of PCa. Surgery (RP) was the primary treatment for 97.9% of the patients, which is due to recruitment procedures for the database. During the follow-up period, BCR occurred in more than a third (35.8%) of the participants. BCR at survey was reported in every fifth patient (19.6%), and 13.5% were under a current treatment. Only a minority of the patients had a clinical level of depressive (7.5%) or anxiety (6.1%) symptoms. More than half of the patients (53.5%) reported a moderate or high perceived severity of the disease experience with high perceived severity for 25.1% of all patients (Table 1).

**Table 1:** Sociodemographic, clinical, and psychosocial characteristics of the study population (*n* = 4252)

	M (SD)	*n*	%
Age at survey (years)	77.4 (6.4)		
≤ 70		558	13.1
>70 ≤ 80		2145	50.5
> 80		1549	36.4
Educational level			
Low		1724	41.3
Intermediate		708	17.0
High		511	12.3
Academic degree		1229	29.4
Partnership			
Yes		3590	85.4
No		615	14.6
Children	1.8 (1.0)		
0		480	11.6
≥ 1		3672	88.4
Age at diagnosis (years)	62.6 (6.2)		
≤ 55		520	12.2
> 55 ≤ 65		2145	50.5
> 65		1587	37.3
Time since diagnosis (years)	14.8 (3.8)		
≤ 10		390	9.2
> 10 ≤ 15		2017	47.4
> 15 ≤ 20		1513	35.6
> 20		332	7.8
Second primary cancer			
Yes		542	12.8
No		3710	87.2
Family history of PCa			
Yes		1637	38.5
No		2615	61.5
PSA level at diagnosis (ng/ml)	10.70 (13.22)		
≤ 4		375	9.5
> 4 ≤ 10		2367	60.2
> 10		1194	30.3
Gleason score			
2–6		1758	50.0
7		1401	39.8
8–10		359	10.2
Grading			
G I		181	4.5
G II		2779	69.5
G III		1040	26.0
Organ-confined stage of disease			
Yes		2926	70.0
No		1256	30.0
Type of primary treatment			
Surgery		4162	97.9
Radiotherapy		62	1.5
Others		28	0.6
Biochemical recurrence during follow-up			
Yes		1520	35.8
No		2732	64,2
Biochemical recurrence at survey			
Yes		831	19.6
No		3420	80.4
Ongoing treatment at survey			
Yes		570	13.5
No		3665	86.5
Clinical level of depressive symptoms (PHQ-2)	0.81 (1.16)		
Yes		300	7.5
No		3716	92.5
Clinical level of anxiety symptoms (GAD-2)	0.71 (1.07)		
Yes		244	6.1
No		3743	93.9
Perceived severity of the disease experience	2.65 (1.00)		
None		562	13.9
Low		1321	32.6
Moderate		1151	28.4
High		1017	25.1

*M*, mean; *SD,* standard deviation; *PCa*, prostate cancer; *PSA*, prostate specific antigen; *RP*, radical prostatectomy; *PHQ-2*, Patient Health Questionnaire-2; *GAD-2*, Generalized Anxiety Disorder-2 scale

Total scores for the BFS were available for 3899 participants (91.7%). A missing data analysis showed that the 353 participants not included were characterized by a higher age (at diagnosis and at survey), lower educational level, no partnership, and a longer time since diagnosis (all *p* ≤ 0.005).

### Benefit finding endorsement and prevalence

Descriptive data for the BFS are shown in Table 2. Strong endorsement of single items ranged from 20.9% (has led me to meet people who have become some of my best friends) to 63.8% (has taught me to adjust to things I cannot change). The mean overall BFS score was *M* **=** 3.14 (SD **=** 1.00). The prevalence of moderate-to-high BF was 59.7%.

**Table 2:** Benefit finding items, mean scores, and strong endorsement frequencies (*n* = 3877–4011)

Item	Having had prostate cancer…	M	SD	% ≥ 4
1	has led me to be more accepting of things	3.32	1.21	50.6
2	has taught me how to adjust to things I cannot change	3.63	1.24	63.8
3	has helped me take things as they come	3.56	1.24	60.2
4	has brought my family closer together	3.08	1.38	43.5
5	has made me more sensitive to family issues	3.17	1.29	46.8
6	has taught me that everyone has a purpose in life	3.00	1.41	42.8
7	has shown me that all people need to be loved	3.54	1.38	59.5
8	has made me realize the importance of planning for my family’s future	3.47	1.38	57.1
9	has made me more aware and concerned for the future of all human beings	2.92	1.32	38.6
10	has taught me to be patient	3.27	1.27	48.5
11	has led me to deal better with stress and problems	3.10	1.26	43.2
12	has led me to meet people who have become some of my best friends	2.31	1.27	20.9
13	has contributed to my overall emotional and spiritual growth	2.75	1.28	31.9
14	has helped me become more aware of the love and support available from other people	3.26	1.32	48.7
15	has helped me realize who my real friends are	2.92	1.45	40.5
16	has helped me become more focused on priorities, with a deeper sense of purpose in life	2.98	1.36	41.3
17	has helped me become a stronger person, more able to cope effectively with future life challenges	3.18	1.37	47.7

*M*, mean; *SD*, standard deviation

### Associations among study variables

Correlational analysis revealed marginal to low significant associations among BF and the psychosocial variables (Table 3). To predict BF, a hierarchical multiple regression analysis was conducted (Table 4). The time since diagnosis was found to have strong intercorrelation with age at diagnosis and age at survey, which led to its removal from the regression analysis model. Step 1 included all sociodemographic variables, accounting for 4.8% of the explained variance. Lower age at diagnosis, higher age at survey, and lower educational level predicted the mean BFS score (all *p* < 0.01). Clinical variables were added in step 2 without increasing the explained variance (ΔR^2^ **=** 0.005). In the last step, the psychosocial variables were included. Higher perceived severity of the disease experience and lower level of depressive symptoms predicted BF, adding 4.6% to the explained variance. In the final model, perceived severity of the disease experience and educational level were the strongest predictors of BF with β coefficients of 0.217 and − 0.181, respectively (both *p* < 0.001). The overall explained variance was 9.6%.

**Table 3:** Correlations between benefit finding and psychosocial variables

	Benefit finding	Perceived severity	Depressive symptoms
Benefit finding	—		
Perceived severity	0.232 ***	—	
Depressive symptoms	0.047 **	0.210 ***	—
Anxiety symptoms	0.081 ***	0.201 ***	0.646***

*Benefit finding*, Benefit Finding Scale; *Perceived severity*, perceived severity of the cancer experience; *Depressive symptoms*, Patient Health Questionnaire-2, *Anxiety symptoms,* Generalized Anxiety Disorder-2 scale; **p < 0.01, ***p < 0.001

**Table 4:** Hierarchical regression analysis for the mean Benefit Finding Scale score (*n* = 3297)

Step		*B*	SE *B*	*β*	Adj. *R*^2^	Δ*R*^2^
1	Sociodemographics				0.048	0.049***
	Age at survey^†^	0.015	0.005	0.095**		
	School education^†^	− 0.125	0.010	− 0.212***		
	Partnership^‡^	0.051	0.050	0.017		
	Children^†^	0.022	0.017	0.022		
	Age at diagnosis^†^	− 0.022	0.005	− 0.135***		
2	+ Clinical variables				0.051	0.005*
	Age at survey^†^	0.013	0.005	0.085**		
	School education^†^	− 0.122	0.010	− 0.208***		
	Partnership^‡^	0.051	0.050	0.017		
	Children^†^	0.024	0.017	0.024		
	Age at diagnosis^†^	− 0.021	0.005	− 0.128***		
	Second primary cancer^‡^	− 0.033	0.051	− 0.011		
	Family history of PCa^‡^	− 0.063	0.035	− 0.030		
	PSA level at diagnosis^†^	− 0.001	0.001	− 0.010		
	Biochemical recurrence during FU^‡^	0.083	0.050	0.039		
	Biochemical recurrence at survey^‡^	− 0.001	0.058	0.000		
	Ongoing treatment at survey^‡^	0.101	0.057	0.034		
3	*+* Psychosocial variables				0.96	0.046***
	Age at survey^†^	0.014	0.005	0.092**		
	School education^†^	− 0.107	0.010	− 0.181***		
	Partnership^‡^	0.041	0.049	0.014		
	Children^†^	0.036	0.016	0.037*		
	Age at diagnosis^†^	− 0.018	0.005	− 0.113***		
	Second primary cancer^‡^	− 0.007	0.050	− 0.002		
	Family history of PCa^‡^	− 0.044	0.035	− 0.021		
	PSA level at diagnosis^†^	− 0.001	0.001	− 0.016		
	Biochemical recurrence during FU^‡^	0.049	0.049	0.024		
	Biochemical recurrence at survey^‡^	0.006	0.057	0.002		
	Ongoing treatment at survey^‡^	0.089	0.056	0.030		
	Depressive symptoms (PHQ-2) ^†^	− 0.044	0.020	− 0.051*		
	Anxiety symptoms (GAD-2) ^†^	0.041	0.022	0.044		
	Perceived severity ^†^	0.220	0.018	0.217***		

*SE*, standard error; *PCa*, prostate cancer; *PSA*, prostate specific antigen; *FU*, follow-up; *PHQ-2*, Patient Health Questionnaire-2; *GAD-2*, Generalized Anxiety Disorder-2 scale; *Perceived severity*, perceived severity of the disease experience

^†^considered continuous variable; ‡scored 0 = no, 1 = yes

*p < 0.05, **p < 0.01, ***p < 0.001

## Discussion

The present study mainly aimed to evaluate the occurrence of benefit finding (BF) in a large sample of long-term PCa survivors in Germany, as knowledge about BF could help promoting positive mental health in the survival course. Furthermore, associations of BF with a broad range of disease-related and psychosocial factors were investigated. Most notably, the perceived severity of the disease experience emerged as the variable most strongly associated with BF, independently of the objective clinical severity of the disease course.

In the present study, moderate-to-high BF occurred in 60% of the PCa survivors after an average of 15 years since diagnosis. Our results suggest that being diagnosed with, treated for and surviving PCa can lead to the perceptions of benefits and to positive life changes in the long term. BF and PTG occurred in 50–93% of cancer survivors in various cancer populations [33, 36, 37]. The large variability of prevalence rates may partly be due to the different cancer entities themselves, but the impact of prevalence definition should also be considered. Although there is no general definition for the occurrence of meaningful, significant BF and PTG in cancer survivors, some studies have defined occurrence of positive changes when at least one benefit was reported, leading generally to higher reported rates [10]. Other studies, however, adopted a narrower definition using a quantitative tool, such as the Benefit Finding Scale (BFS) (i.e., the rate of patients reporting at least a moderate level of BF). Applying the latter definition, similar prevalence rates have been found in survivors of meningioma and colorectal cancer (63% and 64%, respectively) but have not yet been reported in studies considering PCa survivors [6, 32]. Instead, the mean single item and total scores are more frequently reported, allowing sample comparison. The mean total BFS score in our German study population was 3.1, whereas lower mean total BFS scores of 2.1 (unknown follow-up period) and 2.7 (mean of 10.5 months after treatment) in PCa survivors were previously reported in Australia and the USA, respectively [31, 38]. Differences in these levels could perhaps be due to the shorter follow-up periods, as BF and PTG are thought to require time to develop, allowing cognitive and emotional processes to unfold [1, 12, 39]. Additionally, cultural differences are conceivable. Indeed, similar results were found in German long-term colorectal cancer survivors (5-year follow-up) with a prevalence of moderate-to-high BF of 64% and a mean total BFS score of 3.4 [6].

Item endorsement ranking allows qualitatively exploring the appraisal of the different domains of BF. Items reflecting acceptance of and adjustment to the irrevocably changed things in life seem to regularly find the highest endorsement in different cancer populations including those in the present study. These benefits are followed or sometimes preceded by items reflecting enhanced existing relationships with family and friends [31, 32, 40]. In these rankings, the least endorsed item was virtually always “has led me to meet people who have become some of my best friends”, which is the same in the present study. Thus, extending the social network (e.g., fellow patients in a self-support group) seems to be a rather marginal phenomenon after cancer diagnosis, while existing bonds increase in value and appreciation.

Associations between BF and PTG and sociodemographic factors have broadly been evaluated in the past decades. Inconsistent or even contradictory findings have been described as the result of variations in study design and population and the varying analyses of data [1, 40]. Lower educational level and, to a smaller extent, younger age at diagnosis were predictive of BF in the present study population. Lower socioeconomic status, represented here by the educational level as a proxy, has repeatedly been linked to BF and PTG [26, 40, 41]. This association is believed to be due to the patients’ experiences in dealing with disruption and hardships in life [41]. A weak, inverse association with age has been established and is considered to be moderated by higher distress along with greater optimism and openness for change in younger cancer patients [1, 42]. Indeed, even in a mixed sample of older adult long-term cancer survivors (29.5% were PCa survivors; mean age 72.5 years), relatively younger survivors were more likely to report posttraumatic transformation [43]. The effect on BF of age at diagnosis in the present study population was weak. Studies involving PCa survivors could not corroborate this relationship [26, 28, 31]. The low variability of age at diagnosis in patients with PCa could be related to these inconsistent findings, as most men are diagnosed with PCa in ages older than 65 years.

We surveyed BF after a mean time since diagnosis of 14.8 years. To our knowledge, the present study is the first report of BF in PCa survivors with this extended follow-up period. Little is known about the stability of BF over long periods of time. However, when cautiously compared with the lower levels of BF in the short-term PCa survivors mentioned before, our findings seem to support the notion that BF has at least a stable component or could even increase over long periods of time. Accordingly, similar levels of growth were found in breast cancer survivors with 5–15 years postdiagnosis compared with shorter-term survivors in previous studies [15].

Following Tedeschi and Calhoun’s concept of posttraumatic growth, the magnitude of the disrupting “seismic event” must be sufficiently severe to struggle with it and to engage in coping processes [39]. The magnitude or severity of a cancer disease can be considered in objective and self-reported subjective approaches. Objective parameters for the severity of the disease in previous studies included tumor stage, type of primary treatment, adjuvant treatment (like chemotherapy or tamoxifen in breast cancer patients), and impairing side effects. Objective severity, represented by the tumor stage, has been linked to BF and PTG in a (curvi-)linear association, reflecting that the disease should be sufficiently severe—but not too severe—to engage in coping processes leading to the perception of personal growth [4, 41]. Adorno et al. [37] described the endorsement of distinctive domains of BF in patients with localized vs. spread disease. Other studies, however, could often not establish this association [33, 44]. These inconclusive findings could originate from variations in the operationalization of the variables or from the exclusion criteria of many studies (e.g., advanced stages). In studies including PCa survivors, BF and PTG were not associated with the type of primary treatment, differences in the antihormonal therapy regimen, or impaired sexual or urinary functioning [26–28, 40]. Further disease-related factors, such as disease recurrence or current therapy, represent additional stressors in the disease course and might therefore influence the extent of positive changes. However, this relationship did not emerge in the present study. Disease recurrence was defined on a biochemical level (i.e., increased PSA level ≥ 0.2 ng/ml after RP) and does not correspond to a clinical recurrence. Therefore, it might not necessarily reflect the patient’s awareness of the disease progression. However, subsequent current therapy—one can assume is consciously perceived—was not predictive for BF. Thus, patients might not perceive active treatment as a severe adversity, as action is taken to counteract disease recurrence or because they get more resilient over the course of the often-permanent treatment.

Furthermore, a positive family history of PCa or the presence of a second primary cancer may constitute additional factors contributing to distress that have, to our knowledge, not yet been investigated in PCa survivors. Alternatively, they could put one’s current disease into perspective and hereby reduce its threat; for instance, sons of affected fathers could have observed a mild manifestation of the disease. Additionally, the long mean time since diagnosis in our study population could have reduced the impact of the family history on patient’s current distress. Our null findings do not allow to draw firm conclusions about the effect of family history, and its effect should be investigated further in future studies.

High levels of perceived stress, threat, or disease intensity have indeed been established as a promoter of BF and PTG and illustrate the subjective side of the severity of the disease experience [1, 11, 12, 45]. Previous operationalizations of threat and stress in recently diagnosed cancer patients represented their current and future views of the disease (e.g., rating of the subjective likeliness to die of cancer). A retrospective and biographical approach to this factor appeared to us to be more suitable in older long-term survivors. Therefore, we surveyed the perceived severity of the disease experience as an adverse life event asking, “Having had prostate cancer is one of the worst things that happened in my life”*.* It was proven to be the primary predictive factor of BF in the present study population. Hence, our findings confirm and extend previous investigations of this relationship.

## Limitations

There are some limitations to be addressed. First, the cross-sectional design of the present study precludes temporal inferences. Longitudinal studies surveying trajectories of positive changes in the long-term survival course should be considered in future studies. Because of the recruitment procedures for the research project, patients after RP were clearly overrepresented in the present study sample. While previous data did not show an effect for primary treatment regimen in short-term PCa survivors [40], our findings cannot be generalized to radiotherapy or active surveillance/watchful waiting patients. Furthermore, there is another selection bias as our sample included only those patients who survived a very long time after their PCa diagnosis. Moreover, as we did not assess ethnicity, the relevance of sociocultural factors for our results pattern remains unclear. Although several potentially relevant factors were included in the analysis, the explained variance of the regression analysis was low. Other factors with the potential to promote BF and PTG (such as adaptive coping, optimism, resilience, or health-related quality of life) could not be evaluated, and thus, important links could have been missed. Finally, as we did not assess comorbidities or the occurrence of other critical or traumatic life events since PCa diagnosis and treatment, it is unclear whether the development of BF after PCa is also influenced by other, noncancer-related stressors.

## Conclusion and clinical implications

In conclusion, the present study revealed that a large proportion of older (very-) long-term PCa survivors report benefits and growth arising from the disease. Our results suggest that the self-asserted meaning and subjective importance of the disease in the patient’s biography are primarily linked to BF in the survivorship course.

Active exploration of BF and PTG should be considered by the treating clinician independently of the clinical severity of the patient’s disease during the extended follow-up period, as promoting personal growth has been proven to be an important resource for mental health adjustment. Indeed, psychological intervention for PCa survivors showed positive effects on personal growth [38]. Still, it is important to acknowledge that finding benefit and meaning in the cancer experience is not prescriptive but represents an individual journey.

## Data Availability

The authors have full control of all primary data.
